# An economic evaluation of fine-needle cytology as the primary diagnostic tool in the diagnosis of lymphadenopathy

**DOI:** 10.1515/med-2023-0719

**Published:** 2023-06-05

**Authors:** Monica Cavallo, Valeria Ciliberti, Elisabetta Maffei, Bianca Serio, Francesco Sabbatino, Pio Zeppa, Alessandro Caputo

**Affiliations:** Department of Medicine and Surgery, University of Salerno, Baronissi, Salerno, Italy; Department of Oncology, Haematology and Pathology, Pathology Unit, University Hospital of Salerno, Salerno, Italy; Department of Oncology, Haematology and Pathology, Haematology Unit, University Hospital of Salerno, Salerno, Italy; Department of Oncology, Haematology and Pathology, Oncology Unit, University Hospital of Salerno, Salerno, Italy; Department of Medicine and Surgery, University of Salerno, Via Salvador Allende 1, Baronissi, Salerno, Italy; Pathology Unit, University Hospital of Salerno, Salerno, Italy

**Keywords:** fine-needle aspiration cytology, lymphadenopathy, cost-effectiveness, diagnosis

## Abstract

Fine-needle aspiration cytology (FNAC) is commonly used to obtain a pre-surgical pathological diagnosis in many organs, but its cost-effectiveness in lymphadenopathy has not been studied yet. We calculated the cost and diagnostic accuracy of a diagnostic algorithm that uses FNAC as a first-line procedure and compared it to a purely surgical approach in 545 consecutive lymphadenopathies. In 74% of the cases, FNAC alone can obtain a sufficiently detailed diagnosis, avoiding the surgical biopsy. In doing so, the average cost of diagnosis is cut to less than one-third, the patient avoids an invasive procedure and the diagnosis is reached earlier. In conclusion, the systematic use of lymph node-FNAC in the initial assessment of lymphadenopathy is clinically and economically advantageous as it avoids surgical biopsies in cases where cytology can suffice.

## 1 Introduction

Superficial lymphadenopathy is frequently encountered in everyday clinical practice. The exact incidence of lymphadenopathy is unknown but likely underestimated because in many cases it has a benign and self-limited course and is thus not recorded [1,2]. When the clinical presentation is not typical and serological data do not match the clinical context, diagnostic imaging, generally by ultrasound (US), is required [2,3]. When the lymphadenopathy persists, a pathological assessment by lymph node fine-needle aspiration cytology (LN-FNAC) and/or surgical biopsy (SB) is required [2,3].

Traditionally, histological evaluation has been the standard approach to establish the cause of lymphadenopathy [2]. However, several excised LNs turn out to be the site of a reactive process [4]. The histological examination in most of these cases provides only a descriptive evaluation, as different etiologies may show similar morphological features [4]. For this reason, alternative approaches are employed to reduce the number of SBs. LN-FNAC is a minimally invasive procedure with high sensitivity and specificity, particularly in identifying reactive processes, metastases, and recurrences of previously diagnosed lymphoma [5,6]. The association of LN-FNAC to ancillary techniques such as flow cytometry (FC), immunocytochemistry (ICC), polymerase chain reaction (PCR)-based procedures, and fluorescence *in situ* hybridization can improve its accuracy, in particular for the diagnosis of non-Hodgkin lymphoma (NHL) [7]. However, despite these diagnostic improvements, the difficulty in subclassifying NHL on cytological samples remains [6].

Generally, when an LN-FNAC diagnosis is made in conjunction with appropriate ancillary techniques and a proper clinical context, it does not require histopathological confirmation in cases of benign reactive lymphoid hyperplasia, specific infections, recurrent lymphoproliferative disorders, and metastases of known primary tumors [3]. Conversely, histological confirmation of LN-FNAC results is required for the primary diagnosis of Hodgkin’s lymphoma (HL) and NHL, except for specific clinical situations or in some anatomical locations where surgical procedures are contraindicated or not possible [3]. In case of a negative outcome at LN-FNAC, follow-up is indicated; in the case of atypical lymphoid cells of uncertain significance (ALUS), repetition and follow-up are indicated [3]. Follow-up consists of performing clinical surveillance and, in the case of persistence of symptoms, repeating LN-FNAC and/or performing SB. This approach has been systematized in a recent proposal [3]. However, in the absence of worldwide acceptance of these specific indications, to date there are two extreme clinical contexts: one in which LN-FNAC is available, performed, and considered in the clinical management of patients; another, in which LN-FNAC is not performed or not accepted as a diagnostic tool and histological evaluation is the only diagnostic procedure for the morphological diagnosis of lymphadenopathy. In fact, in the institutions where LN-FNAC is utilized as the primary procedure to diagnose lymphadenopathy, it is sufficient to establish a diagnosis of negativity, metastases, and lymphoma relapse in the majority of cases, except for clinically very suspect lymphadenopathies or those which do not regress at the follow-up. In the same institutions, subsequent excisional SB is reserved to primary NHL and HL, metastases from an unknown neoplasm, and to all the cases in which LN-FNAC does not achieve a definitive diagnosis of benignity. Conversely, in the institutions where LN-FNAC is not available or not performed, all the lymphadenopathies suspected to be malignant undergo SB and histological examination. Numerous studies evaluating the sensitivity and specificity of LN-FNAC are available, as well as studies describing rare entities or complex diagnoses, but the cost of the procedure as a primary diagnostic approach has been evaluated for metastases only [8].

This study aims to compare the costs of the two methods to achieve a definitive diagnosis in cases of lymphadenopathy. For this purpose, we calculated the costs of a diagnostic algorithm relying on LN-FNAC as a screening procedure and compared it to an algorithm employing excisional SB directly to assess whether the former can reduce the overall cost of diagnosis and the number of SBs performed.

## 2 Materials and methods

### 2.1 Data

This study retrospectively analyzed the data regarding a series of consecutive clinically suspicious lymphadenopathies that were investigated using LN-FNAC in an 18-month period at our institution.

Patients’ data (age, gender, LN size, and US features), diagnostic procedures, final diagnoses, and follow-up data were extracted from the electronic health records of the hospital. Data concerning the performance of LN-FNAC, US data, cytological diagnoses, and histological results (when available) were extracted from the database of our institution. Data concerning follow-up, LN-FNAC repetitions when performed, and clinical evaluations of the patients who did not undergo surgery were collected with a minimum follow-up of 12 months.

### 2.2 FNAC

All LN-FNACs were performed by one of two experienced cytopathologists. They were also performed with US assistance both for unpalpable and palpable LNs to reach the target and the most representative areas of the LN avoiding the hilum when represented, as previously described [9,10]. Rapid on-site evaluation (ROSE) was performed in all the cases to assess the adequacy of the sample, the need for immediate repetition for inadequate cases, or for harvesting additional materials. Moreover, ROSE enabled us to manage the material for specific ancillary techniques choosing appropriate technical supports as previously described, such as formalin for cell blocks and phosphate-buffered saline for FC [3,9,11,12]. Ancillary techniques were performed when necessary and chosen on the basis of clinical data and ROSE cytological features, as suggested by the Sydney System [3]. They were performed also on histological samples according to general and specific algorithms and protocols [3,13,14].

LN-FNACs were diagnosed according to the recently published Sydney System [3] as benign (reactive), atypical (lymphoid) cells of uncertain significance (AUS/ALUS), suspicious, or malignant (HL, NHL, metastasis). As all cases were evaluated with ROSE and inadequate/insufficient cases were immediately repeated, there were no inadequate cases in the present study. According to the previously cited algorithm [3], ALUS cases were also limited in the present series because ROSE indicated the need for FC, which was performed and allowed for the reclassification of most cases. ALUS cases in which FC was not contributive were referred to clinical follow-up and underwent repetition or SB in case of persistence of the lymphadenopathy, according to the clinician’s judgment. All cases diagnosed as suspicious, as well as a portion of positive cases (NHL, HL, and metastases from unknown or undetermined primary site), underwent excisional SB of the corresponding LN whenever possible. Positive cases at LN-FNAC were not followed by SB in cases of relapse of lymphoma and metastasis of known primary or in which ICC identified the tumor of origin.

### 2.3 Calculation of costs

The costs of the two possible algorithms, namely LN-FNAC possibly followed by SB versus upfront SB, were calculated by evaluating the reimbursements that the Italian National Health System (NHS) grants to public hospitals and accredited private institutions for LN-FNAC and LN-SBs, respectively. These data were obtained from the rate tables of the Campania region; the reimbursements in these rate tables refer to the ICD-9-CM classification. The reimbursements included costs of equipment and materials required to perform the respective procedures, personnel costs, and costs of the structures. The costs of any complications or prolongation of any hospitalization were not included, and the costs of the clinical follow-up for negative LN-FNAC were not included as well as they were difficult to estimate. The impact that these procedures have on the patient’s quality of life was also not considered, both in terms of lost working days and in terms of non-calculable moral costs. As far as the costs of ancillary techniques are concerned, it was almost impossible to compare their costs in LN-FNAC and SB because those utilized in the former may be different from those in the latter both in type and number of tests. Furthermore, the Italian NHS does not reimburse single ancillary tests, except for specific molecular tests. Therefore, starting from the assumption that ancillary technique costs are similar either if performed on cytological, cell block, or histological samples and that they are equally performed in the two techniques, in this study, the impact of ancillary techniques on the two compared procedures was not calculated.

The costs of the overall LN-FNAC diagnostic evaluation were calculated by adding the reimbursements of each procedure carried out to reach a diagnosis. In particular, the costs in the case of the use of the LN-FNAC in the initial evaluation of clinically suspicious lymphadenopathies have been calculated by adding the cost of any repeated LN-FNAC or any SB to the cost of the first LN-FNAC. The cost of managing lymphadenopathy by SB only, without using LN-FNAC, was calculated assuming that an SB had been performed in all cases.

### 2.4 Decision model

In the present study, the Markov decision model was adopted to calculate the cost of a definitive diagnosis in clinically suspicious lymphadenopathies [15]. This model is a multistate transient model in which patients make transitions among various health states at different rates for extended periods (cycles) [22]. This model has already been utilized in other studies dealing with cost analysis of thyroid FNAC [16,17,18]. As far as LNs are concerned, a study was performed on the cost utility of sentinel LN-FNAC in the staging of oral cancer, utilizing the Markov model [19]. In our case, the Markov decision model has been applied to the management and care process of patients with clinically suspicious lymphadenopathies, in relation to the initial cytological result. In this analytical decision-making model, the first cycle started always with an LN-FNAC. Each patient, based on the LN-FNAC results, was put into one of four transient states, namely benign, undetermined (ALUS), suspicious, and malignant. This model always assumed that each patient fell into one of these four states. The events of interest for the diagnosis (follow-up, LN-FNAC repetition, SB) were considered events that ended the previous cycle and started the next one. At the end of each cycle, each patient could persist in the same state of health as the previous cycle or move to a new state. Patients who received SB after LN-FNAC entered the state corresponding to that defined by the biopsy; for example, a lymphadenopathy resulted benign to LN-FNAC that carried out an SB, which turned out to be malignant, went into the malignant state. Patients performing a second LN-FNAC go into the state corresponding to the result of the second LN-FNAC. For example, a lymphadenopathy resulted benign to the first LN-FNAC that carried out a second LN-FNAC, which turned out to be malignant, went into the malignant state. Patients who were followed-up clinically without repeating LN-FNAC or performing an SB remained in the same state; for example, a patient with a lymphadenopathy with a benign first LN-FNAC result, who no longer performed another LN-FNAC or SB on suspected lymphadenopathy, remained in the benign state.

For positive and suspect cases at LN-FNAC, the diagnostic process was considered concluded when a final diagnosis was achieved with SB or, in cases where it was sufficient, by LN-FNAC. For negative and ALUS cases, the diagnostic process was considered concluded either when the clinical follow-up and the clinician’s judgment confirmed the LN-FNAC diagnosis of benignity or, lacking this clinical reassurance when SB was performed.

### 2.5 Statistical analysis

Cases classified as positive or suspect at LN-FNAC for which SB and the following histological examination confirmed that the malignancy was considered true positives. Cases classified as negative or ALUS at LN-FNAC for which follow-up or SB confirmed benignity were considered true negatives. Cases with suspect or positive LN-FNAC diagnosis and negative SB histology were considered false positives. Negative or undetermined (ALUS) cases to cytology with positive SB histology were considered false negatives.


**Ethical approval:** All procedures performed in studies involving human participants were in accordance with the ethical standards of the institutional and/or national research committee and with the 1964 Helsinki Declaration and its later amendments or comparable ethical standards.

## 3 Results

In the 18-month period examined, 545 LN-FNACs were performed on 535 lymphadenopathies (535 first LN-FNACs + 10 repetitions). Subsequently, 131 (24%) underwent SB.

The series include 293 females (54.8%) and 252 males. The average age at the time of LN-FNAC was 56.4 (±18.4) years. The mean diameter of the lymph nodes was 21.7 mm with a standard deviation of 9.5 mm.

### 3.1 Baseline data

As previously reported, the model applied for the management of suspected lymphadenopathy refers to the Markov model. The first step of the model for each of the enrolled patients corresponded to the result of LN-FNAC, which was malignant in 275 cases (51.40%), benign in 240 (44.86%), suspect in 15 (2.81%), and undetermined (ALUS) in 5 (0.93%). Because ROSE was employed, no cases were inadequate. These percentages represent the initial probabilities of the model.

Of the 275 malignant LN-FNACs, 167 were positive for metastasis, 98 for NHL, and 10 for HL. In the case of metastatic lymphadenopathies, an SB was performed in seven cases. In the remaining 160 cases, LN-FNAC and ICC were sufficient to obtain an accurate diagnosis and identify the primary tumor; therefore, there was no need to perform an SB. In cases that were positive for lymphoproliferative disorders (HL, NHL), SB was performed in 94 cases; the remaining 14 cases represented those in which surgery was contraindicated or not needed (e.g., recurrence of already known lymphoproliferative disease). Of these 94, in 1 case, it was necessary to repeat the SB because a wrong lymph node had been removed and repetition confirmed the LN-FNAC diagnosis of malignancy. Therefore, all these cases were considered true positives.

Regarding the 240 cases in which the LN-FNAC gave a benign result, 220 lymphadenopathies regressed at clinical follow-up and did not need further diagnostic investigations. In 10 cases, LN-FNAC was repeated within 2 months because of the persistence of symptoms and in 10 cases an SB was performed because the clinical and US features were highly suggestive of malignancy. Of the 10 lymphadenopathies that repeated a second FNAC within 2 months for the persistence of the symptomatology, 9 obtained a result of negativity (benign) and 1 of suspicious. Of the 9 lymphadenopathies for which benignity was confirmed with a second FNAC, only in 1 case SB was required and confirmed benignity (true negative); in all the other cases, the lymphadenopathy regressed and SB was not necessary. The single lymphadenopathy that obtained a suspect result at the second FNAC was subjected to an SB, which carried out a diagnosis of negativity and was considered a false positive. The ten lymphadenopathies classified as benign at the first FNAC that presented clinical features suspicious for malignancy were subjected to SB and, of these, three received a histological diagnosis of malignancy (false negatives) and seven of benignity (true negatives).

All 15 LN-FNAC suspicious for lymphoproliferative process underwent SB and were positive at histological evaluation (true positives).

Of the five LN-FNACs with undetermined outcome (ALUS), two did not continue the diagnostic procedure because clinical features suggested benignity, and three underwent SB. Of these, one was found to be positive for metastasis (false negative) and two were benign at histology (true negatives).

### 3.2 Markov model results

At the end of the model, 395 cases (73.8%) did not require an SB for histological control or a second LN-FNAC and so persisted in the first state of Markov model, identified by the result of the first LN-FNAC. In 174 (44.05%) of these 395 cases, LN-FNAC gave a diagnosis of metastasis from a known primary tumor, identified the primary tumor when unknown, or diagnosed a relapse from a known NHL or HL. Of the other 222 cases that did not require further investigation except follow-up, 220 (55.02%) were identified at LN-FNAC as benign and 2 (0.50%) as ALUS. The cases that required SB in addition to the first LN-FNAC were 131 (24.48%); as the first LN-FNAC was clinically not sufficient and did require histological control, the corresponding patients moved to the following state of the Markov model corresponding to that defined by the SB histological result. Of these 131 cases, at first LN-FNAC, 12 (7.87%) were identified as benign, 3 (2.36%) as ALUS, 15 (11.81%) as suspicious, and 101 (77.09%) as malignant. The malignant cases included primary NHL or HL, which required SB histological confirmation and metastases in which LN-FNAC could not identify the primary tumor. In ten cases (1.86%), instead of the SB, a second LN-FNAC was performed; so, in these cases, the patients moved into a new state of Markov model. In eight cases (1.49%), a second LN-FNAC was sufficient and so the corresponding patient persisted in the second state corresponding to that defined by the second LN-FNAC. In the remaining two cases (0.37%), it was necessary to perform also an SB so these two cases moved to a third state of Markov model corresponding to that resulting from the SB. After three cycles, 54.58% of the cohort received a final diagnosis of malignancy.

**Figure A1:**
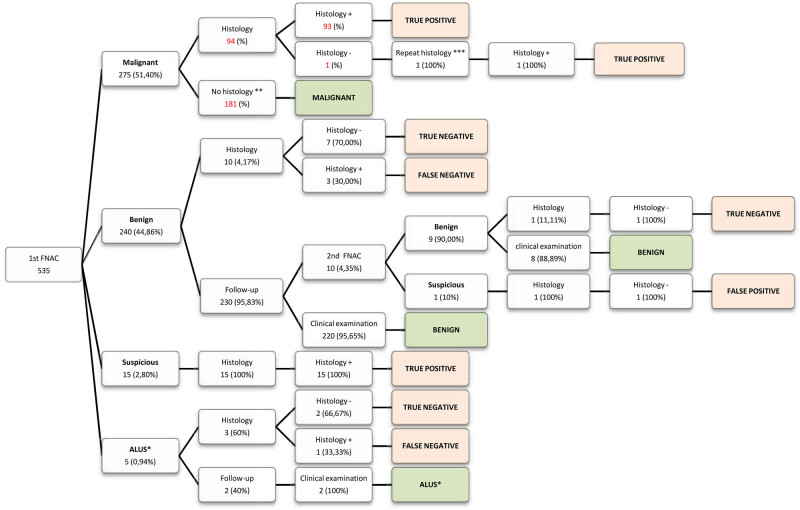
Flow chart displaying the iterations of the Markov model in our series of 545 lymphadenopathies. Abbreviations: ALUS, atypical lymphoid cells of undetermined significance; FNAC, fine-needle aspiration cytology.

To summarize, 131 out of 535 lymphadenopathies studied required an SB and were classified as malignant in 120 (91.60%) cases and benign in 11 (8.39%) cases. The whole model is shown in Figure A1.

### 3.3 Diagnostic performance

The diagnostic performance of LN-FNAC was calculated. In our whole series, LN-FNAC showed a sensitivity of 98.6%, a specificity of 99.6%, a positive predictive value of 99.7%, and a negative predictive value of 98.4%. By calculating these indices only in cases that underwent LN-SB, we obtain a sensitivity of LN-FNAC of 96.7%, a specificity of 90.9%, a positive predictive value of 99.14%, and a negative predictive value of 71.42%.

### 3.4 Assessment of costs

According to the criteria reported above, the average cost for the execution of an LN-FNAC, which includes the execution of the US-assisted LN-FNAC, ROSE, basic technical processing, ancillary techniques if any, interpretation, diagnosis, and reporting was €129.50 per case.

The average cost of the SB as reimbursed to the hospitals by the Italian NHS, which includes the cost for the day surgery hospitalization, the surgical procedure, the histological examination, and reporting, was €1666.64.

Applying the Markov model for three cycles, it was calculated that using LN-FNAC as the first procedure, the estimated cost of diagnosis per patient was €540.01. This cost accounts for €129.50 that represents the cost of the first LN-FNAC, performed in all 535 lymphadenopathies, plus the second LN-FNAC when performed (10 cases) plus the cost of an SB when performed (131 cases). Most of the costs for the diagnosis (75.57%) were due to the SBs requested in cases where the LN-FNAC for different reasons was not sufficient to reach a definitive diagnosis. This cost amounted to €408.09 per patient.

In the case of undetermined (ALUS) or suspicious outcome at the first LN-FNAC, the diagnosis per patient costed €1796.14 and €1129.48, respectively. In the case of benignity or malignancy, the cost per patient was €218.23 and €741.61, respectively.

The estimated cost to get a diagnosis without using LN-FNAC would have been €1666.64 per patient. This cost was calculated considering performing an SB in all patients with suspected lymphadenopathy (535 cases), without a previous LN-FNAC.

Comparing the two costs, we calculated an average saving using LN-FNAC of €1126.63 per patient. An algorithm employing LN-FNAC as a first screening procedure costs per patient, on average, about one-third (32%) of an algorithm that employs SB directly.

## 4 Discussion

FNAC examination has proved to be a simple, accurate, and safe method for the preoperative diagnosis of numerous clinical conditions including lymphadenopathies. Numerous studies have analyzed the qualities of LN-FNAC including diagnostic accuracy, but only a few studies have evaluated the potential impact of LN-FNAC in the clinical management of lymphadenopathies from an economic point of view. The present study aims to determine the cost of diagnosing lymphadenopathies using LN-FNAC and to compare these costs to those of SB.

**Figure 1:**
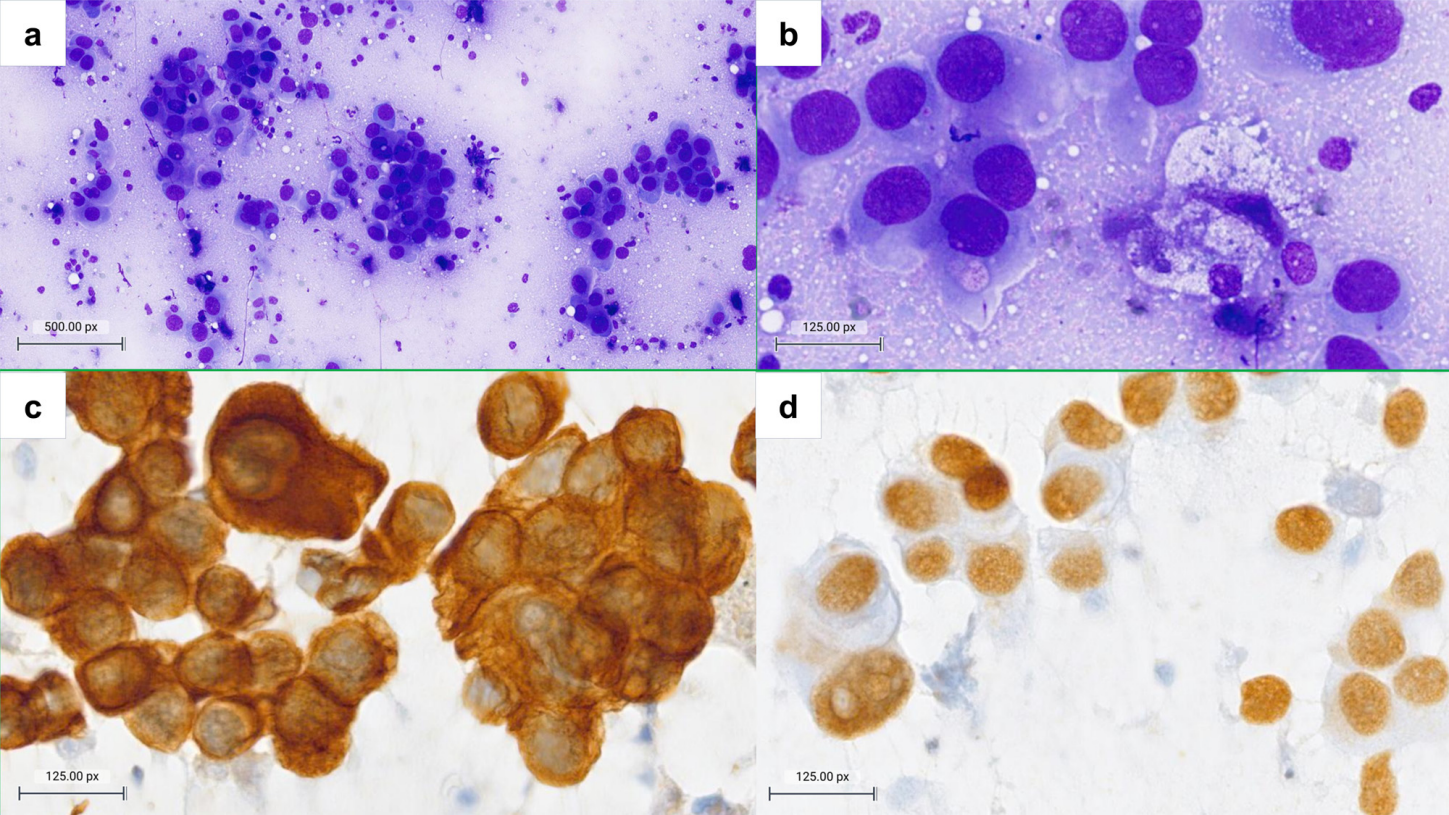
Lymph nodal metastasis from pulmonary adenocarcinoma confirmed with ICC. (a and b) Cytomorphology shows infiltration of the lymph node by a diffuse population of atypical epithelioid cells with dense, finely vacuolated cytoplasms and large nuclei with anisokaryosis. Immunocytochemical positivity for cytokeratin 7 (c) and TTF-1 (d) confirmed the diagnosis. (a) Diff-Quik, 100×, (b) Diff-Quik, 400×, and (c and d) diaminobenzidine ICC, 400×.

There are several scenarios in which LN-FNAC gives a definitive diagnosis and thus avoids an LN-SB and is economically convenient. For example, cytology can confirm a recurrence, relapse, or new localization of a known disease. A cytological diagnosis of malignant, positive for metastasis of a known primary tumor, with immunocytochemical confirmation, is often enough for the clinician to treat the patient accordingly without the need for an excisional biopsy (Figure 1) [20]. Such an issue can also happen with a benign cytological diagnosis, for example in a lymphadenopathy in a patient with known sarcoidosis [21]. But LN-FNAC can also perform a diagnosis that stands on its own: this is the case of infectious lymphadenopathy when an additional material is saved for ancillary techniques such as PCR for the identification of the specific agent, e.g., tuberculous lymphadenopathy [3].

In the present series, 414 LN-SBs were avoided by performing 545 LN-FNACs; in other words, for every four LN-FNACs performed, just one was followed by LN-SB, while the other three were sufficient to reach a diagnosis.

Besides, there are benefits of LN-FNAC that cannot be quantified in economic terms and are due to the general qualities of FNAC as the lower invasiveness and shorter delay from the request to the execution and the report when compared to LN-SB. As a matter of fact, in our institution, like in many others, LN-FNAC is usually performed within 24 h of the request of the clinician, whereas weeks can pass between the request of LN-SB and its performance.

Analyzing the Markov model applied in this study, all the lymphadenopathies completed the diagnostic process within 3 cycles; however, 403 out of 535 (75%) received a final diagnosis within the first cycle; 533 out of 535 (99.6%) received a final diagnosis within the second cycle, and only 2 cases required the third cycle. This rate is comparable to or higher than those obtained in other similar studies [19]. Despite that, some authors are skeptical about the use of LN-FNAC in the initial evaluation of lymphadenopathies for the possible danger of false-negative results, as they could lead to a diagnostic delay. In the present series, using LN-FNAC in association with ancillary techniques, a false-negative rate of 3.4% was calculated. This rate is similar to those reported in other studies in which LN-FNAC technical procedures and ancillary techniques are mostly standardized [22,23]. Moreover in the calculation of the false-negative rate, similarly to other studies [22,23], patients who did not undergo surgery but were clinically followed-up were not considered because histology was considered the gold standard, so we obtained a false-negative rate higher than real. The applied Markov model considers certain not only the histological confirmation of the LN-FNAC diagnosis but also the permanence in the same state at the end of the third cycle. Therefore, confirming the first LN-FNAC through a second one and/or clinical evaluation, the rate of false-negative using LN-FNAC in association with ancillary techniques was of 1.4%, hence much lower than the percentage obtained considering only cases with a histological control. Part of the skepticism toward LN-FNAC comes from studies that considered only LN-SB, and, in this way, overestimated the percentage of false negatives. The present study has not evaluated the possible diagnostic delay caused by LN-FNAC as the first diagnostic approach. Analyzing retrospectively all the lymphadenopathies that needed histological confirmation (primary HL and NHL, ALUS, and suspect cases that remain in the same diagnostic state after LN-FNAC repetition), the median delay from the first LN-FNAC to the LN-SB was 20 days (interquartile range, 18.25). This delay other than extra costs in economic terms certainly caused additional emotional distress to the patients. Nonetheless, we believe that this delay is partially diluted and attenuated by the time necessary to perform concomitant unspecific or specific pre-treatment tests and procedures, namely laboratory, imaging, and cardiology tests that are generally requested in symptomatic and asymptomatic patients in most cases of lymphadenopathy; moreover, in cases of primary or relapsed highly suspicious NHL or HL, blood product transfusion, antibiotics, hydration, lumbar puncture, and bone marrow biopsy are also requested [24], and represent a time in which LN-FNAC can be performed and/or repeated also in concomitance with other diagnostic procedures, without delaying the performance of LN-SB. Moreover, even LN-SB may require repetition because of technical problems or in case the targeted LN is not representative of the pathology. Finally, we believe that the emotional burden on patients who still undergo LN-SB after LN-FNAC (25%) is largely compensated by the patients who avoid LN-SB altogether (75%).

Finally, other than economic reasons, it has to be considered that, as LN-FNAC performance needs fewer prerequisites than SB, it can be programmed and performed earlier and more easily than SB.

Based on these considerations, it is possible to assume that the use of LN-FNAC does not increase the time necessary to obtain a diagnosis but instead reduces it allowing a diagnosis to be made in a less advanced stage of the disease. The Markov model has been successfully used to evaluate therapeutic and diagnostic procedures, albeit with specific limitations for the latter. For instance, in the study by Borget et al. [18] performed on thyroid FNAC, final surgery was performed for different reasons (aesthetic, therapeutic, and diagnostic); because it was difficult to attribute the cost to one or another, in their basal model, the cost of surgery was applied to all the patients with unsatisfactory cytopathologic results who had undergone surgery regardless of the final histopathologic result [18]. In the case of lymphadenopathy, SB is often a diagnostic procedure and only seldom therapeutic. Therefore, ideally, any ALUS, suspect, or positive LN-FNAC for which histological evaluation reveals a reactive process should be considered a false positive. Conversely, any negative LN-FNAC that a following SB reveals to be an NHL, HL, or a metastasis should be considered a false negative. However, taking into account these considerations, in institutions where LN-FNAC represents the first step of a structured algorithm for the diagnosis of lymphadenopathies, the only side effects of a LN-FNAC false negative or false positive are a slight delay on the final diagnosis or the performance of an SB that would have been equally performed without LN-FNAC.

A limitation of our study is that the calculation of the cost of diagnosis in the absence of FNAC is estimated. In estimating the overall cost, the cost of the histological evaluation was applied in all cases without considering any contraindications to the surgical intervention and without considering that the clinician in some cases could have delayed the histological evaluation. Nonetheless, it is worth considering that costs can be only estimated and not fully and accurately measured since in addition to consumables, FNAC and ancillary techniques require good quality instrumentation (US machine, flow cytometer) and its rent and repair costs, as well as operators and their training and salary costs. The SB and histological examination too carry additional expenses that are hard to accurately quantify and estimate beforehand. Finally, some costs are shared by FNAC and SB, such as blood tests and imaging.

Core biopsy (CB) represents a reliable alternative to SB; we use this procedure in our institution albeit with specific limitations related to LN size, mobility, anatomical site, and case-specific risks. For these reasons, CB is not routinely used and has not been considered in the present study. Furthermore, some studies have demonstrated the superiority of CB compared to FNAC in obtaining a definitive diagnosis of lymphadenopathies [25]; others have also obtained comparable results regarding SB and CB to the point of considering CB a first-line method in the evaluation of lymphadenopathies [25,26,27,28]. CB is less expensive than SB but more expensive than FNAC. However, one limitation of CB is its stringent criteria that limit its usage to only a portion of cases. In this sense, the costs should be re-evaluated considering the immediate execution of the CB in conjunction or as an alternative to the FNAC where possible. Further research is needed in this direction.

One additional exciting new perspective is the application of digital pathology to LN-FNAC. For example, a digital slide can be sent for consultation in a few seconds to multiple colleagues simultaneously, and without risk of slide damage [29]. Furthermore, digital specimens can be used to develop computer-aided diagnosis tools that can help the pathologist in all kinds of tasks, from quantification of immunohistochemistry (e.g., counting the percentage of Ki67-positive cells) to directly identifying and highlighting rare malignant cells in several smears, or interpreting a full slide and obtaining a tentative diagnosis [30,31,32].

## 5 Conclusions

This study explored the usage of LN-FNAC as the first step in the diagnosis of lymphadenopathies. We show that it is economically convenient, in addition to being safe and accurate. In 74% of cases, a diagnosis is reached by LN-FNAC alone, avoiding an SB. In doing so, the average cost of diagnosis is cut to less than one-third, the patient avoids an invasive procedure, and the diagnosis is reached earlier [3,4,33]. The systematic use of LN-FNAC in the initial assessment of lymphadenopathies is clinically and economically advantageous as it avoids surgical biopsies in cases where cytology can suffice.

## References

[1] Bazemore A, Smucker DR. Lymphadenopathy and malignancy. Am Fam Phys. 2002;66:2103–10. PMID: 12484692. 12484692

[2] Mohseni S, Shojaiefard A, Khorgami Z, Alinejad S, Ghorbani A, Ghafouri A. Peripheral lymphadenopathy: Approach and diagnostic tools. Iran J Med Sci. 2014;39(2 Suppl):158–70. PMID: 24753638. PMC3993046 24753638

[3] Al-Abbadi MA, Barroca H, Bode-Lesniewska B, Calaminici M, Caraway NP, Chhieng DF, et al. A proposal for the performance, classification and reporting of lymph node fine needle aspiration cytopathology: The Sydney system. Acta Cytol. 2020;64(4):306–22. 10.1159/000506497. 32454496

[4] Moor JW, Murray P, Inwood J, Gouldesbrough D, Bem C. Diagnostic biopsy of lymph nodes of neck, axilla and groin: rhyme, reason or chance? Ann R Coll Surg Engl. 2008;90(3):221–5. 10.1308/003588408X242105. PMC2430458 18430337

[5] Nasuti JF, Yu G, Boudousquie A, Gupta P. Diagnostic value of lymph node fine needle aspiration cytology: An institutional experience of 387 cases observed over a 5-year period. Cytopathology. 2000;11:18–31. 10.1046/j.1365-2303.2000.00208.x. 10714372

[6] Lioe TF, Elliott H, Allen DC, Spence RA. The role of fine needle aspiration cytology (FNAC) in the investigation of superficial lymphadenopathy; uses and limitations of the technique. Cytopathology. 1999;10:291–7. 10.1046/j.1365-2303.1999.00183.x. 10588346

[7] Dunphy CH, Ramos R. Combining fine-needle aspiration and flow cytometric immunophenotyping in evaluation of nodal and extranodal sites for possible lymphoma: A retrospective review. Diagn Cytopathol. 1997;16:200–6. 10.1002/(sici)1097-0339(199703)16:3<200::aid-dc2>3.0.co;2-j. 9099538

[8] Van Akkooi AC, Voit CA, Verhoef C, Eggermont AM. Potential cost-effectiveness of US-guided FNAC in melanoma patients as a primary procedure and in follow-up. Ann Surg Oncol. 2010;17(2):660–4. 10.1245/s10434-009-0807-8. PMC2816814 19921338

[9] Paksoy N, Yazal K. Organization of a fine-needle aspiration clinic with cytopathologist-radiologist cooperation: An alternative way to Rome. J Ultrasound Med. 2021 Jan;40(1):183–5. 10.1002/jum.15370. PMID: 32529682. 32529682

[10] Caleo A, Vigliar E, Vitale M, Di Crescenzo V, Cinelli M, Carlomagno C, et al. Cytological diagnosis of thyroid nodules in Hashimoto thyroiditis in elderly patients. BMC Surg. 2013;13(Suppl 2):S41. 10.1186/1471-2482-13-S2-S41. Epub 2013 Oct 8 PMID: 24266923; PMCID: PMC3851014. PMC3851014 24266923

[11] Caputo A, Ciliberti V, D’Antonio A, D’Ardia A, Fumo R, Giudice V, et al. Real-world experience with the Sydney System on 1458 cases of lymph node fine needle aspiration cytology. Cytopathology. 2022;33(2):166–75. 10.1111/cyt.13079. Epub ahead of print. PMID: 34817104. 34817104

[12] Troisi J, Colucci A, Cavallo P, Richards S, Symes S, Landolfi A, et al. A serum metabolomic signature for the detection and grading of bladder cancer. Appl Sci. 2021;11(6):2835. 10.3390/app11062835.

[13] Sabbatino F, Scognamiglio G, Liguori L, Marra A, Anniciello AM, Polcaro G, et al. Peritumoral immune infiltrate as a prognostic biomarker in thin melanoma. Front Immunol. 2020 Sep 29;11:561390. 10.3389/fimmu.2020.561390. PMID: 33117345; PMCID: PMC7550791. PMC7550791 33117345

[14] Scognamiglio G, D’Antonio A, Rossi G, Cavazza A, Camerlingo R, Pirozzi G, et al. CD90 expression in atypical meningiomas and meningioma metastasis. Am J Clin Pathol. 2014 Jun;141(6):841–9. 10.1309/AJCP8Y0EBQMKWQEI. PMID: 24838329. 24838329

[15] Sonnenberg FA, Beck JR. Markov models in medical decision making: A practical guide. Med Decis Mak. 1993;13(4):322–38. 10.1177/0272989X9301300409. 8246705

[16] Kuo EJ, Wu JX, Zanocco KA. Cost effectiveness of immediate biopsy versus surveillance of intermediate-suspicion thyroid nodules. Surgery. 2018;164(6):1330–5. 10.1016/j.surg.2018.06.003. 30055789

[17] Heller M, Zanocco K, Zydowicz S, Elaraj D, Nayar R, Sturgeon C. Cost-effectiveness analysis of repeat fine-needle aspiration for thyroid biopsies read as atypia of undetermined significance. Surgery. 2012;152(3):423–30. 10.1016/j.surg.2012.05.038. 22938902

[18] Borget I, Vielh P, Leboulleux S, Allyn M, Iacobelli S, Schlumberger M, et al. Assessment of the cost of fine-needle aspiration cytology as a diagnostic tool in patients with thyroid nodules. Am J Clin Pathol. 2008;129(5):763–71. 10.1309/H86KM785Q9KBWPW5. 18426737

[19] van der Linden N, Flach GB, de Bree R, Uyl-de Groot CA. Cost-utility of sentinel lymph node biopsy in cT1-T2N0 oral cancer. Oral Oncol. 2016;53:20–6. 10.1016/j.oraloncology.2015.11.011. 26631953

[20] Acanfora G, Iaccarino A, Dello Iacovo F, Pisapia P, De Luca C, Giordano C, et al. A roadmap for a comprehensive diagnostic approach to fine needle cytology of lymph node metastases. Cytopathology. 2022 Nov;33(6):668–77. 10.1111/cyt.13172. Epub 2022 Sep 5, PMID: 35986701, PMCID: PMC9826057. PMC9826057 35986701

[21] Tana C, Donatiello I, Caputo A, Tana M, Naccarelli T, Mantini C, et al. Clinical features, histopathology and differential diagnosis of sarcoidosis. Cells. 2021 Dec 26;11(1):59. 10.3390/cells11010059. PMID: 35011621, PMCID: PMC8750978. PMC8750978 35011621

[22] Gharib H, Goellner JR, Johnson DA. Fine-needle aspiration cytology of the thyroid. A 12-year experience with 11,000 biopsies. Clin Lab Med. 1993;13(3):699–709. 8222583

[23] Hafez NH, Tahoun NS. Reliability of fine needle aspiration cytology (FNAC) as a diagnostic tool in cases of cervical lymphadenopathy. J Egypt Natl Canc Inst. 2011;23(3):105–14. 10.1016/j.jnci.2011.09.009. 22776815

[24] Kane G, Fleming C, Heneghan H, McCartan D, James P, Trueick R, et al. False-negative rate of ultrasound-guided fine-needle aspiration cytology for identifying axillary lymph node metastasis in breast cancer patients. Breast J. 2019;25(5):848–52. 10.1111/tbj.13402. 31197915

[25] Accordino MK, Wright JD, Vasan S, Neugut AI, Hillyer GC, Hershman DL. Factors and costs associated with delay in treatment initiation and prolonged length of stay with inpatient EPOCH chemotherapy in patients with hematologic malignancies. Cancer Invest. 2017;35(3):202–14. 10.1080/07357907.2016.1276186. PMC5700415 28165774

[26] Ryu YJ, Cha W, Jeong WJ, Choi SI, Ahn SH. Diagnostic role of core needle biopsy in cervical lymphadenopathy. Head Neck. 2015;37(2):229–33. 10.1002/hed.23580. 24375555

[27] Allin D, David S, Jacob A, Mir N, Giles A, Gibbins N. Use of core biopsy in diagnosing cervical lymphadenopathy: A viable alternative to surgical excisional biopsy of lymph nodes? Ann R Coll Surg Engl. 2017;99(3):242–4. 10.1308/rcsann.2016.0353. PMC5450284 27917669

[28] Han F, Xu M, Xie T, Wang JW, Lin QG, Guo ZX, et al. Efficacy of ultrasound-guided core needle biopsy in cervical lymphadenopathy: A retrospective study of 6,695 cases. Eur Radiol. 2018;28(5):1809–17. 10.1007/s00330-017-5116-1. 29188372

[29] Caputo A, D’Antonio A. Digital pathology: The future is now. Indian J Pathol Microbiol. 2021 Jan-Mar;64(1):6–7. 10.4103/IJPM.IJPM_247_20. PMID: 33433402. 33433402

[30] Caputo A, D’Antonio A, Memoli D, Sabbatino F, Altieri V, Zeppa P. Ki67 in Gleason pattern 3 as a marker of the presence of higher-grade prostate cancer. Appl Immunohistochem Mol Morphol. 2021 Feb 1;29(2):112–7. 10.1097/PAI.0000000000000835. PMID: 32107350. 32107350

[31] Sanghvi AB, Allen EZ, Callenberg KM, Pantanowitz L. Performance of an artificial intelligence algorithm for reporting urine cytopathology. Cancer Cytopathol. 2019 Oct;127(10):658–66. 10.1002/cncy.22176. Epub 2019 Aug 14. PMID: 31412169. 31412169

[32] Marini N, Marchesin S, Otálora S, Wodzinski M, Caputo A, van Rijthoven M, et al. Unleashing the potential of digital pathology data by training computer-aided diagnosis models without human annotations. NPJ Digit Med. 2022 Jul 22;5(1):102. 10.1038/s41746-022-00635-4. PMID: 35869179; PMCID: PMC9307641. PMC9307641 35869179

[33] Ghirardelli ML, Jemons V, Gobbi PG. Diagnostic approach to lymph node enlargement. Haematologica. 1999;84(3):242–7. 10189390

